# The plasminogen protein is associated with high myopia as revealed by the iTRAQ-based proteomic analysis of the aqueous humor

**DOI:** 10.1038/s41598-021-88220-9

**Published:** 2021-04-22

**Authors:** Kai Wen, Xianfeng Shao, Yahong Li, Yaoling Li, Yongtao Li, Qing Wang, Ruihong Su, Lujie Zhang, Yang Cai, Jing Sun, Yan Zhang

**Affiliations:** 1 Tianjin Key Laboratory of Retinal Functions and Diseases, Tianjin Branch of National Clinical Research Center for Ocular Disease, Eye Institute and School of Optometry, Tianjin Medical University Eye Hospital, No. 251, Fukang Road, Nankai District, Tianjin, 300110 China; 2 Tianjin Children’s Hospital, Tianjin, China

**Keywords:** Refractive errors, Vision disorders, Medical research

## Abstract

To explore the pathogenesis of high myopia (HM) using quantitative proteomics. The aqueous humor of patients with simple nuclear cataract and nuclear cataract complicated with HM (hereinafter referred to as “C” and “HM” groups, respectively) were collected. The isobaric tags for relative and absolute quantitation (iTRAQ)-based liquid chromatography–tandem mass spectrometry (LC–MS/MS) proteomics approach was employed to explore differentially expressed proteins (DEPs). Bioinformatics was used to interpret the proteomic results. Furthermore, the plasminogen (PLG) protein was confirmed by enzyme-linked immunosorbent assay (ELISA) as the candidate biomarker for HM through a receiver operating characteristic curve analysis. The study showed 32 upregulated and 26 downregulated proteins. The gene ontology analysis demonstrated that 58 DEPs corresponded to 325 biological processes, 33 cell components, and 45 molecular functional annotations. The Kyoto Encyclopedia of Genes and Genomes analysis showed that the upregulated DEPs were highly enriched in the coagulation and complement cascades, consistent with the gene set enrichment analysis. Our data suggested that some DEPs might be hallmarks of the development of HM. ELISA confirmed that the PLG expression levels were significantly upregulated in HM. This was a new study investigating alterations in protein levels and affected pathways in HM using iTRAQ-based quantitative proteomics. Our study provided a comprehensive dataset on overall protein changes and shed light on its potential molecular mechanism in human HM.

## Introduction

High myopia (HM), defined as myopia exceeding 6.00 diopters (D) or axis length ≥ 26 mm, significantly increases the risk of pathologic ocular changes^1^. A study pointed out that at present, 163 million people are suffering from HM, accounting for 2.7% of the world's total population, and that in the future, HM will show a significant increase in prevalence globally, affecting nearly 1 billion people by 2050^2^. In addition to environmental factors, genetic predisposition seems to play a role in the development of HM. In recent years, the development of new technologies, such as the genome-wide association analysis, has advanced studies on candidate genes in many traditional HM loci, such as MYP1 and MYP2. Several new gene loci, such as MYP20 and MYP21, have been discovered^3,4^. However, the mechanism of pathological damage in the eyes with HM is unclear.

Proteomics provides a new perspective and method for identifying the pathogenesis of diseases. The proteomic analysis of the aqueous humor in various ocular diseases, including diabetic retinopathy, primary open-angle glaucoma, cataract, and myopia, revealed protein biomarkers of the mechanisms, diagnosis, and novel therapies for the diseases^5–9^. The aqueous humor is an intraocular fluid that supplies nutrients and removes metabolic wastes from avascular tissues of the eye. It has been used to identify the correlation between altered protein expressions and the prognosis of many eye diseases^5,8–10^. However, there is no proteomic study on the mechanism of eye damage caused by HM. Proteomics can demonstrate high-throughput quantitative protein expressions, which provide new theoretical bases and solutions for discovering disease mechanisms. The isobaric tags for relative and absolute quantification (iTRAQ) technology developed by AB SCIEX, USA is an accurate quantitative technique for in vitro isotopic labeling. It uses up to eight isotope reagents to label the N-terminal or lysine side chain of peptides. A high-resolution mass spectrometer can simultaneously analyze the protein expression of these eight samples. It has been a promising quantitative technique in proteomics in recent years.

In the present study, we performed the iTRAQ-based quantitative proteomic analysis of the aqueous humor of patients with nuclear cataract complicated with HM and simple nuclear cataract to identify functional proteins associated with the pathogenesis of HM. Additionally, we performed enzyme-linked immunosorbent assay (ELISA) to confirm the iTRAQ results. Our findings are likely to enhance our understanding of the mechanisms of HM in humans.

## Materials and methods

### Aqueous humor samples

In this prospective study, patients with simple nuclear cataract and nuclear cataract complicated with HM (hereinafter referred to as “C” and “HM” groups, respectively) who first visited the Tianjin Medical University Eye Hospital (Tianjin, China) were enrolled, and their aqueous humor samples were collected during cataract surgery. Inclusion criteria were nuclear cataract and an axis length ≥ 26 mm. Exclusion criteria were other eye and systemic diseases, such as hypertension, diabetes, coronary heart disease, and autoimmune disease. Fourteen aqueous humor samples of eleven patients, including seven samples from each group, were selected in the study. Table 1 shows patient characteristics. Informed consent was obtained from all participants of this study. The collection and preparation of the samples are performed by the same skilled surgeon as follows. 2 eye drops of oxybuprocaine hydrochloride 0.4% (Beinuoxi, Santen Pharmaceutical Co., Ltd, Osaka, Japan) were applied to the eye before rinsing twice with 0.5% povidine iodine (Anerdian, LikangDisinfection Co., Ltd, Shanghai, China). After that, the conjunctival sac was irrigated with a balanced salt solution before the aqueous humor sample collection. To avoid blood and other ocular surface contaminants, the sample was collected using a 1 mL tuberculin syringe with a 30 gauge needle at the corneal limbus prior to any other entry into the eye under the surgical microscope. Then, all samples were collected into a cryopreservation tube and stored at − 80 °C until use. This study was approved by the Human Research Ethics Committee of the Tianjin Medical University Eye Hospital and adhered to the tenets of the Declaration of Helsinki.

**Table 1:** Patient characteristics.

Samples	Gender		Ages		Axial		Eye	
	C	HM	C	HM	C	HM	C	HM
1	F	M	73	60	23.69	29.20	OD	OS
2	F	M	59	78	23.67	29.69	OD	OS
3	F	M	73	59	23.17	29.95	OS	OD
4	F	M	58	59	25.42	29.71	OS	OS
5	F	M	68	78	22.92	29.56	OS	OS
6	M	F	61	78	22.92	29.56	OS	OS
7	M	F	66	70	23.97	26.29	OS	OD
*P*			0.578		< 0.01			
Mean			65.43 ± 6.29	67.71 ± 8.50	23.93 ± 0.87	28.69 ± 1.61		

*C* simple nuclear cataract, *HM* nuclear cataract complicated with high myopia, *F* female, *M* male.

### Sample preparation

We lysed 50 µL aqueous humor with 350 µL 8 M urea buffer (8 M urea [Sigma], 100 mM triethylammonium bicarbonate [TEAB; Sigma], 1 × protease inhibitor [Roche]) at room temperature for 5 min. The lysis products were sonicated on ice and centrifuged at 16,000 × g for 10 min at 15 °C. After removing precipitates, the protein concentration was measured by bicinchoninic acid kits (Solabio). For each sample, 100 µg total protein was transferred into a new 1.5-mL tube, and 10 mM DL-dithiothreitol (Sigma) was added and incubated at 37 °C for 1 h. Subsequently, 40 mM iodoacetamide (Sigma) was added and incubated in dark for 1 h at room temperature. For balance, 400 µL 100 mM TEAB was added to a 10 kDa filter (Sartorius). The sample was transferred onto the filter and centrifuged at 14,000 × g for 20 min at 4 °C. The sample was washed thrice with 400 µL of 100 mM TEAB and collected using a new tube with 100 µL of 100 mM TEAB. For digestion, 2 µg sequencing grade modified trypsin was used for digestion at 37 °C for 12–16 h. The resulting peptides were dried with SpeedVac (Thermo Fisher, SPD1010). iTRAQ 8-plex kits were used to label the peptides by following the manufacturer’s protocols (Table 2). To normalize protein expression, the quality control sample, pooled from an equal amount of aqueous humor of 14 patients, was labeled with mass tag 113. Small high pH reversed-phase peptide fractionation columns were used to fractionate samples with gradually increasing acetonitrile concentrations (6%, 9%, 12%, 15%, 18%, 21%, 25%, 30%, and 35%). The nine fractions were combined into six (6% + 25%, 9% + 30%, 12% + 35%, 15%, 18%, and 21%). Peptides were dried and resuspended with 0.1% formic acid.

**Table 2:** The experimental design of iTRAQ experiments.

	113	114	115	116	117	118	119	121
iTRAQ 8plex kit1	QC	C	HM	C	HM	C	HM	C
iTRAQ 8plex kit2	QC	HM	C	HM	C	HM	C	HM

### LC–MS/MS analysis

Samples were analyzed with the TripleTOF 6600 mass spectrometer (AB SCIEX, USA) coupled with the ekspert nano-LC 415 nanoflow LC system (AB SCIEX, USA). Fractionated peptides were loaded onto an in-house made trap (100 μm × 2 cm; particle size, 3 μm; SunChrom, USA) with mobile phase A (v/v/v, 2% acetonitrile, 0.1% formic acid, and 97.9% water) and separated with a linear gradient of mobile phase B (v/v/v, 0.1% formic acid, 97.9% acetonitrile, and 2% water) on the analytical column (150 μm × 15 cm; particle size, 1.9 μm; SunChrom, USA). The effective elution gradient was 5–35% for 1.5 h at a flow rate of 600 nL/min. The accumulation times for time-of-flight MS and product ions were 0.25 and 0.04 s, respectively. The mass ranged from 300 to 1500 m/z. The charge state ranged from + 2 to + 5, and mass tolerance was less than 50 ppm. We monitored 60 ions/cycle, and the ion exclusion time was 16 s. The fragmentation energy was adjusted using iTRAQ reagents, and a high sensitivity mode was used for scanning.

### Protein identification and quantification

For each iTRAQ set, raw files from eight fractions were combined to search a human database (Swiss human database, released in March 2019, comprising 20,425 entries) with MaxQuant software version 1.6.4.0. Dynamic modifications included N-acetylation, oxidation of methionine, and cysteine carbamidomethylation as the fixed modification. All reporter ions were applied for quantification. The false discovery rate (FDR) was set at 1% for both peptide spectrum matches and proteins. At least two peptides per protein were chosen for quantification. The mass tolerance was 0.1 Da for precursor ions and 50 ppm for fragment ions.

### Statistical and bioinformatic analyses

Data were normalized with quality control between two iTRAQ sets, and median normalization was applied to adjust the error among samples. Student's *t*-test was applied to calculate *p*-values. Screened proteins with a *p *value < 0.05 and fold change > 1.5 were considered to be significantly expressed. The gene ontology (GO) and KEGG pathway^11,12^ enrichment analysis was processed through cluster Profiler, an R package. The adjusted *p*- and *q*-values were both < 0.05. Protein–protein interactions (PPIs) were analyzed with a web tool called STRING, version 11.0, and visualized with Cytoscape software version 3.7.0. Proteins were classified according to their annotated functions. The gene set enrichment analysis (GSEA) was executed with GSEA software version 3.0 (FDR < 0.25, normalized *p* value < 0.05). The principal component analysis was conducted with the stats package and visualized with the factoextra R package.

### ELISA and receiver operating characteristic (ROC) curve analysis

The target protein plasminogen (PLG) was chosen for verification of differential expressions using ELISA. The Human Plasminogen ELISA kit (Elabscience Biotechnology Co., Ltd., E-EL-H2102c) was used. ELISA assays were performed according to the manufacturer's protocols, and samples (HM group, 14 samples; C group, 10 samples) were run in duplicate. The absorbances of the standards and samples were determined by spectrophotometry at 450 nm with a microplate reader. Statistical analyses were performed with GraphPad Prism version 6.0. Student's *t*-test was applied for comparisons of quantitative data, and the ROC curve analysis with GraphPad Prism version 6.0 was performed to evaluate the sensitivity and specificity of the protein PLG.

## Results

### Quantitative proteomic analysis

**Figure 1:**
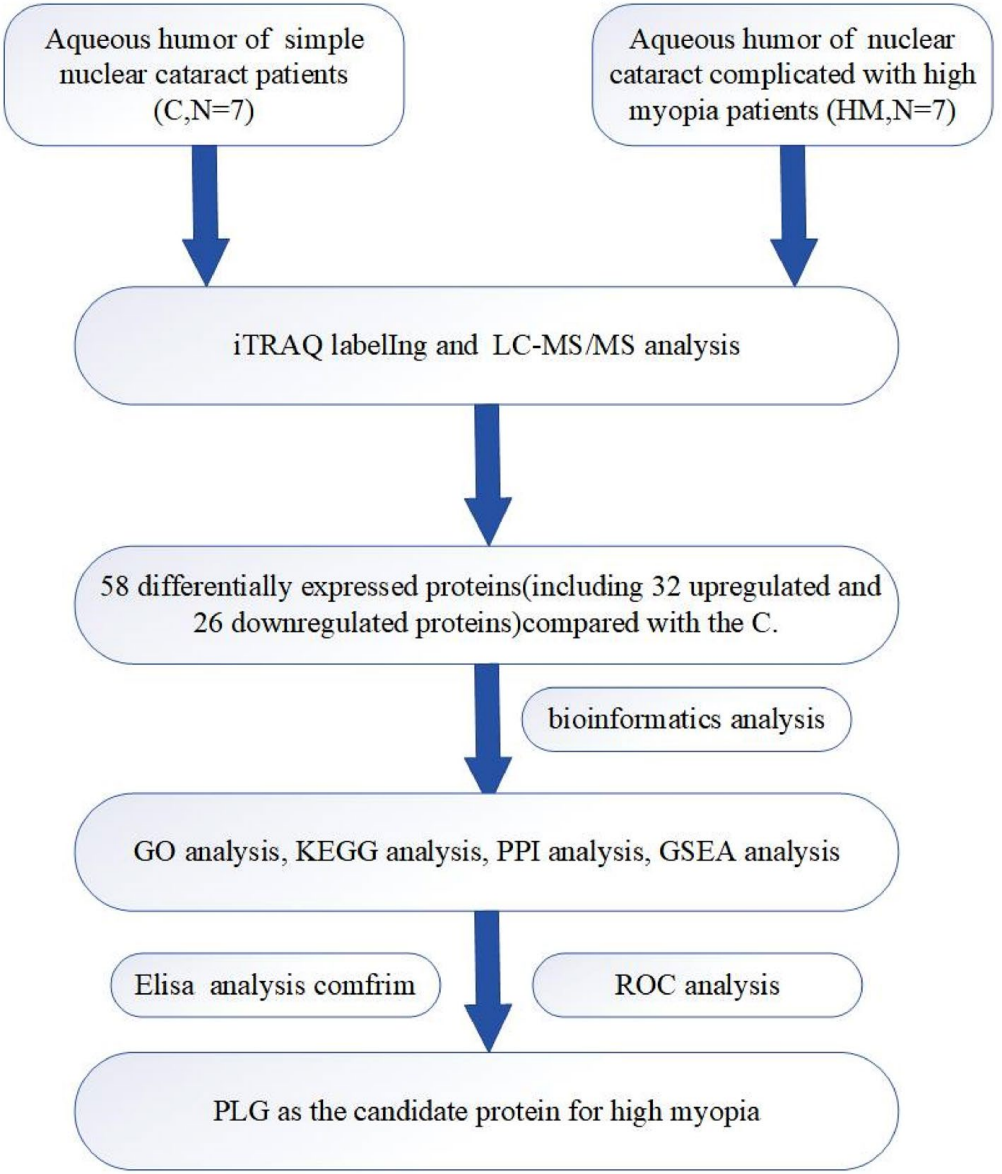
The experimental plan of the present study. iTRAQ was applied to identify the differentially expressed proteins in C and HM. PLG was candidate protein for pathogenesis of high myopia.

Aqueous humor proteins that showed differential accumulation between C and HM groups were identified and analyzed with LC–MS/MS. In the proteomic analysis, a total of 211 unique corresponding proteins were quantified. Among proteins showing significant changes (*p* < 0.05) in abundance, we used proteins with values of an abundance fold change > 1.5 to identify those with a significant difference in abundance between the C and HM groups. Figure 1 shows the experimental design of the whole study.

**Figure 2:**
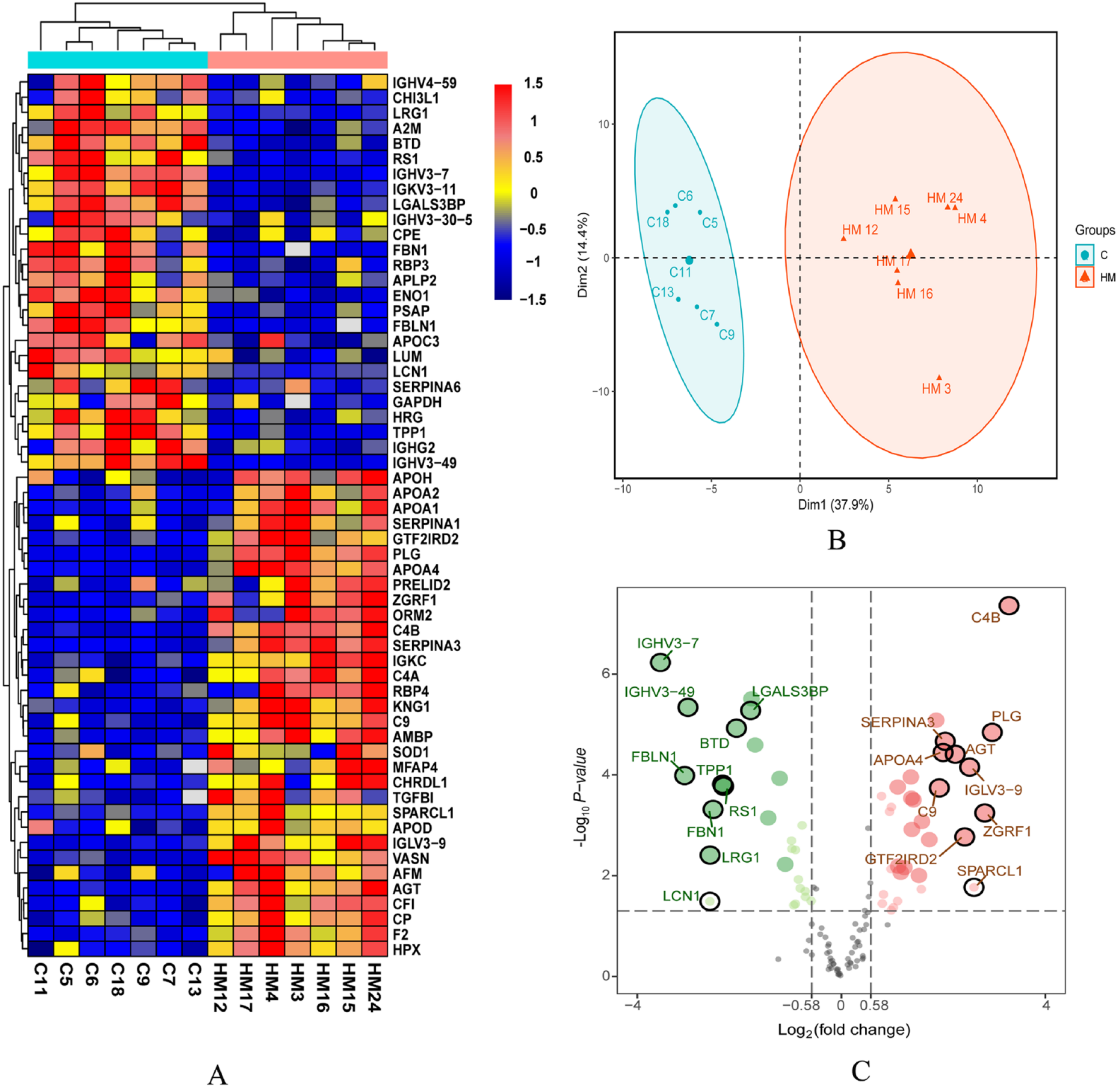
(**A**) Hierarchical cluster analysis of the differentially expressed proteins (Columns represent samples, rows represent proteins, and the shorter the distance of cluster branches represents the higher the similarity of proteins). Red represents up-regulated proteins and blue represents down-regulated proteins. (**B**) The result of principal component analysis (PCA), indicating that there were significant differences in protein expression between the C and HM. (**C**) Differential protein volcano plot showed that these were distributed in two different quadrants (The logarithm of the fold change of each quantifiable protein was taken as base 2, and the negative logarithm of the *P* value was taken as base 10). Horizontal axis represents the fold change (log2 value) of the differential protein, and vertical axis represents the *P* value (− log10 value). Gray dots represent proteins with insignificant differences, red dots represent up-regulated proteins, and blue dots represent down-regulated proteins. The top10 protein was selected to display.

The study showed 58 differentially expressed proteins (DEPs), including 32 upregulated and 26 downregulated proteins (Table 3). The hierarchical cluster analysis showed that the expression pattern of DEPs differed between the two groups (Fig. 2A). Consistently, the results of the principal component analysis and volcano plot showed that DEPs were distributed in two different quadrants (Fig. 2B,C). Among these proteins, the five most upregulated proteins were P0C0L5 (complement), P00747 (plasminogen), Q86YA3 (protein ZGRF1), Q14515 (high endothelial venule protein), and A0A075B6K5 (immunoglobulin lambda variable 3–9), and five most downregulated proteins were P01780 (immunoglobulin heavy variable 3–7), P23142 (fibulin-1), A0A0A0MS15 (immunoglobulin heavy variable 3–49), P31025 (lipocalin-1), and P02750 (rho-GTPase-activating protein LRG1). The significant difference in abundance indicated the complex changes of proteome in HM under the refractive state.

**Table 3:** Quantitative proteomic analysis of 58 differentially expressed proteins, including 32 upregulated and 26 downregulated proteins.

Accession	Gene symbol	Sequence length	Description	*p* value
**Downregulated proteins**				
P01780	IGHV3-7	117	Immunoglobulin heavy variable 3-7	5.89E−07
P23142	FBLN1	703	Fibulin-1	1.03089E−04
A0A0A0MS15	IGHV3-49	119	Immunoglobulin heavy variable 3-49	4.56645E−06
P31025	LCN1	176	Lipocalin-1	0.0324
P02750	LRG1	347	Leucine-rich alpha-2-glycoprotein	0.0039
P35555	FBN1	2871	Fibrillin-1	0.0005
O14773	TPP1	563	Tripeptidyl-peptidase 1	0.0002
O15537	RS1	224	Retinoschisin	0.0002
P43251	BTD	543	Biotinidase	1.18505E−05
Q08380	LGALS3BP	585	Galectin-3-binding protein	5.27E−06
P04433	IGKV3-11	115	Immunoglobulin kappa variable 3-11	3.09E−06
P06733	ENO1	434	Alpha-enolase	2.55032E−05
P07602	PSAP	524	Prosaposin	0.0007
P01023	A2M	1474	Alpha-2-macroglobulin	0.0001
Q06481	APLP2	763	Amyloid-like protein 2	0.0060
P04196	HRG	525	Histidine-rich glycoprotein	0.0020
P51884	LUM	338	Lumican	0.0029
P36222	CHI3L1	383	Chitinase-3-like protein 1	0.0385
P04406	GAPDH	335	Glyceraldehyde-3-phosphate dehydrogenase	0.0364
P01859	IGHG2	326	Immunoglobulin heavy constant gamma 2	0.0031
P16870	CPE	476	Carboxypeptidase E	0.0119
P0DP03	IGHV3-30-5	117	Immunoglobulin heavy variable 3-30-5	0.0178
P10745	RBP3	1247	Retinol-binding protein 3	0.0010
P01825	IGHV4-59	116	Immunoglobulin heavy variable 4-59	0.0204
P02656	APOC3	99	Apolipoprotein C-III	0.0263
P08185	SERPINA6	405	Corticosteroid-binding globulin	0.0318
**Upregulated proteins**				
P02790	HPX	462	Hemopexin	0.0002
P05090	APOD	189	Apolipoprotein D	0.0358
P02749	APOH	345	Beta-2-glycoprotein 1	0.0236
P00450	CP	1065	Ceruloplasmin	0.0005
P43652	AFM	599	Afamin	0.0074
Q8N945	PRELID2	189	PRELI domain-containing protein 2	0.0485
P0C0L4	C4A	1744	Complement C4-A	0.0004
P00441	SOD1	154	Superoxide dismutase [Cu–Zn]	0.0427
P02652	APOA2	100	Apolipoprotein A-II	0.0319
P01834	IGKC	107	Immunoglobulin kappa constant	0.0002
P01009	SERPINA1	418	Alpha-1-antitrypsin	0.0065
Q15582	TGFBI	683	Transforming growth factor-beta-induced protein ig-h3	0.0086
P02753	RBP4	201	Retinol-binding protein 4	0.0069
P05156	CFI	583	P05156	0.0001
P01042	KNG1	644	Kininogen-1	0.0003
P19652	ORM2	201	Alpha-1-acid glycoprotein 2	0.0012
P02760	AMBP	352	Protein AMBP	0.0003
Q9BU40	CHRDL1	456	Chordin-like protein 1	0.0099
P55083	MFAP4	255	Microfibril-associated glycoprotein 4	0.0186
P00734	F2	622	Prothrombin	0.0008
P02647	APOA1	267	Apolipoprotein A-I	0.0019
Q6EMK4	VASN	673	Vasorin	8.22652E−06
P02748	C9	559	Complement component C9	0.0002
P06727	APOA4	396	Apolipoprotein A-IV	3.60425E−05
P01011	SERPINA3	423	Alpha-1-antichymotrypsin	2.15865E−05
P01019	AGT	485	Angiotensinogen	3.89348E−05
Q86UP8	GTF2IRD2	994	General transcription factor II-I repeat domain-containing protein 2A	0.0017
A0A075B6K5	IGLV3-9	115	Immunoglobulin lambda variable 3–9	7.01549E−05
Q14515	SPARCL1	664	SPARC-like protein 1	0.017036259
Q86YA3	ZGRF1	2104	Protein ZGRF1	0.0006
P00747	PLG	810	Plasminogen	1.43342E−05
P0C0L5	C4B	1744	Complement C4-B	4.38E−08

### Bioinformatic analysis of DEPs

#### Data quality control

**Figure 3:**
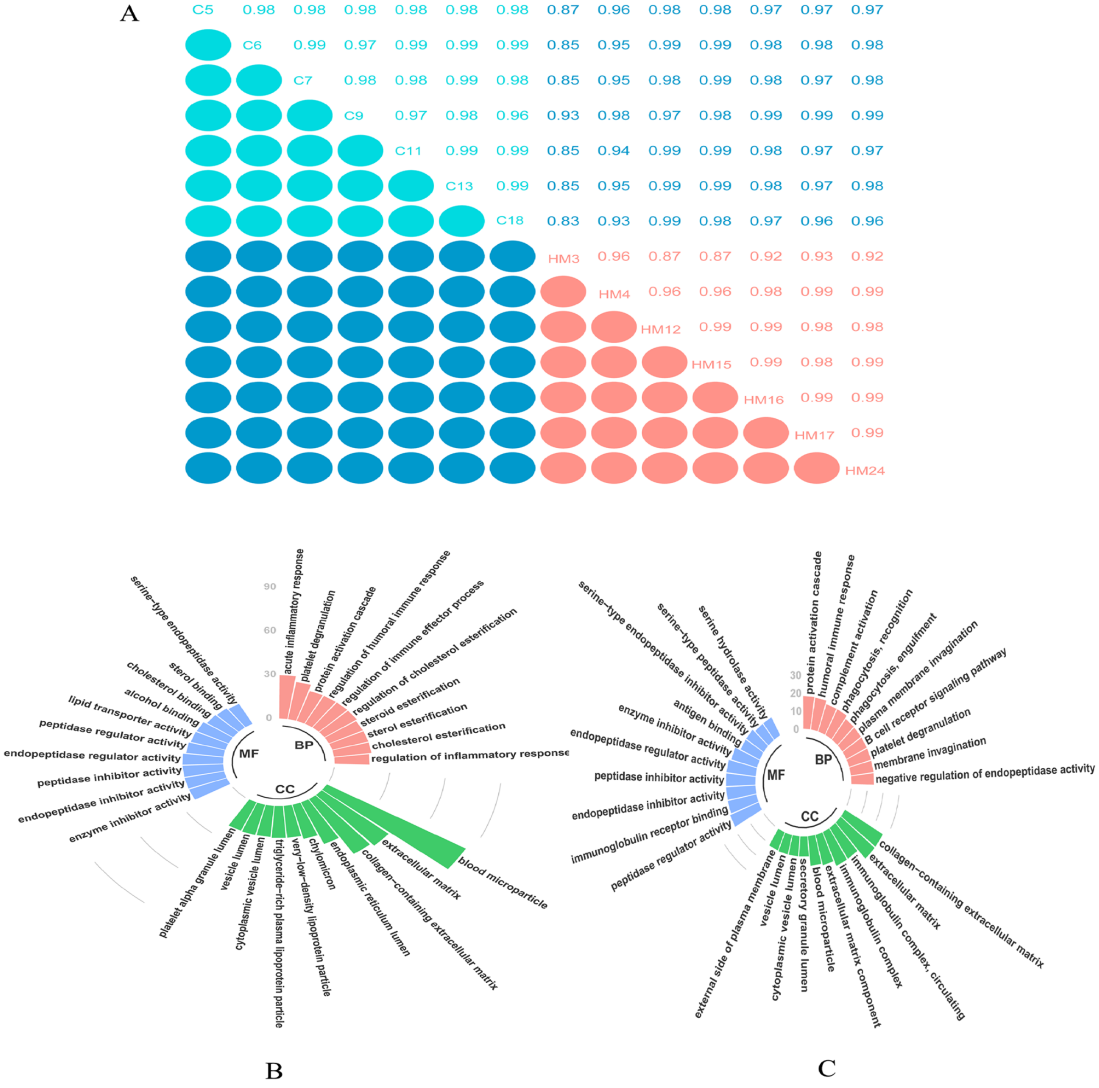
(**A**) Pearson correlation coefficient between samples showed overall data quality. (**B**) and (**C**) Gene Ontology (GO) classification of the upregulated differentially expressed proteins and downregulated differentially expressed proteins, respectively. Proteins were classified into three main categories: biological process, cellular component, and molecular.

Figure 3A shows the Pearson correlation coefficients of the samples. The correlation coefficients in control and HM groups were above 0.96 and 0.87, respectively, indicating high reproducibility of the data.

#### GO analysis

To evaluate the major biological processes that can be perturbed by HM, the GO enrichment analysis was performed on DEPs. The results showed that 58 DEPs were involved in 403 functional annotations. Figures 3B (DEPs involved) and C (downregulated DEPs involved) present the enrichment analysis results. The most significant annotation functions were the acute inflammatory response, platelet degranulation, and protein activation cascade for upregulated proteins and protein activation cascade, humoral immune response, and complement activation for downregulated proteins.

#### KEGG analysis

**Figure 4:**
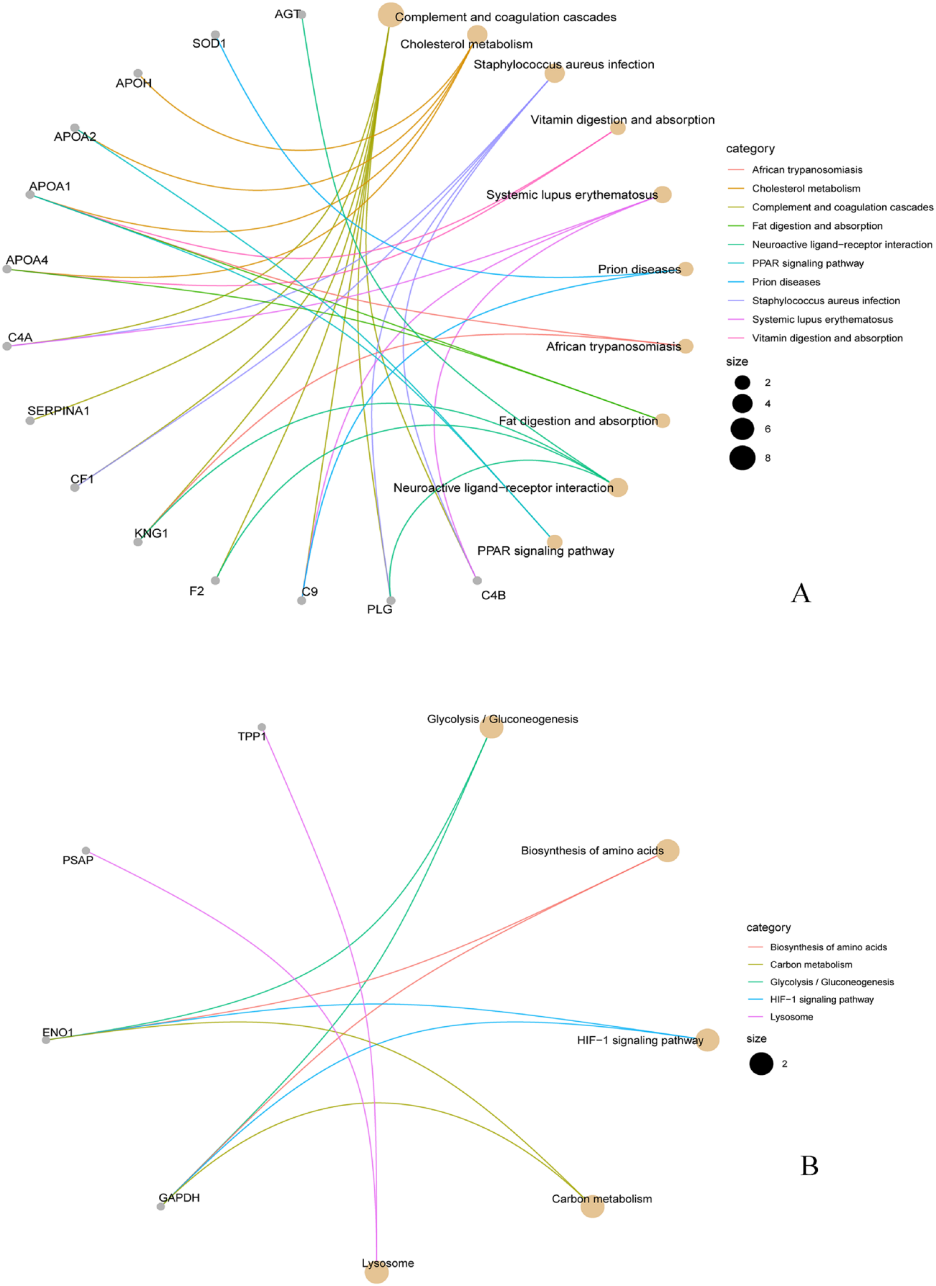
(**A**) and (**B**) Kyoto Encyclopedia of Genes and Genomes (KEGG) enrichment analysis of the upregulated differentially expressed proteins and downregulated differentially expressed proteins, respectively (Select the top 10 by the value of p.adjust).

To analyze the main biochemical processes and associated pathophysiological pathways affected by HM, a KEGG pathway^11,12^-based enrichment analysis was conducted to determine the DEPs in the aqueous humor. The affected pathways upregulated the proteins involved in the complement and coagulation cascades and cholesterol metabolism (Fig. 4A) and downregulated the proteins involved in glycolysis/gluconeogenesis, biosynthesis of amino acids, and HIF-1 signaling pathway (Fig. 4B). A total of eight DEPs were confirmed as key signal molecules of these pathways, including C4B (complement), PLG (plasminogen), C9 (complement component), F2 (thrombin), KNG1 (kininogen-1), CF1 (cleavage stimulation factor subunit 2), SERPINA1 (alpha-1-antitrypsin), and C4A (complement). GO showed that DEPs were mainly associated with inflammation and humoral immunity, and the major proteins involved (C4B, PLG, C9, F2, CFI, SERPINA1, and C4A) coincided with the complement coagulation cascade, implying that DEPs may participate in the process of eye damage caused by HM through the immune process involved in the complement system.

**Figure 5:**
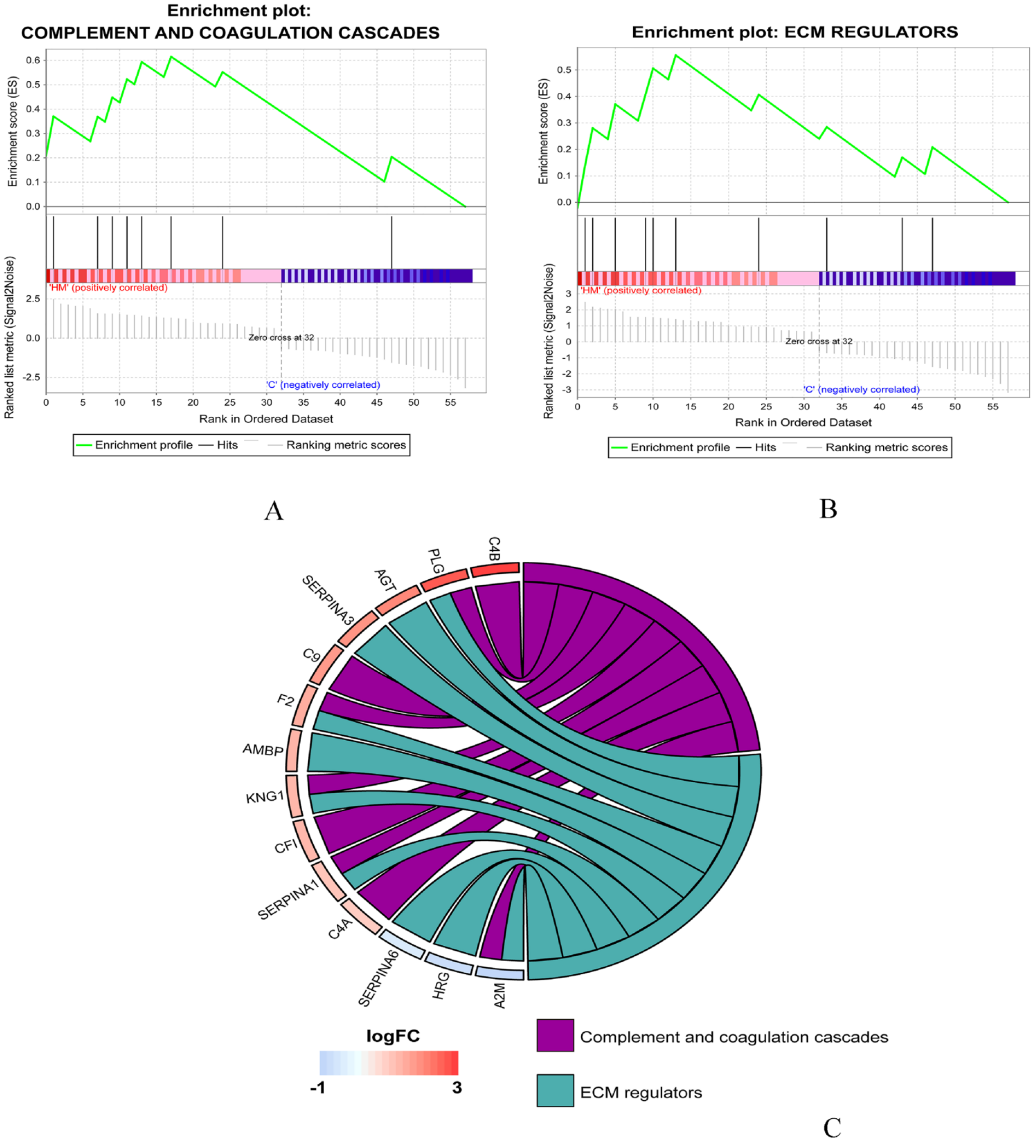
Gene set enrichment analysis showed that the two classical signaling pathways of complement coagulation cascade (**A** and **C**) and extracellular matrix regulator (**B** and **C**), were enriched and upregulated.

Moreover, we performed GSEA (*p* < 0.05, FDR < 0.25) on DEPs and found that the two classical signaling pathways, the complement coagulation cascade and extracellular matrix regulator, were enriched and upregulated (Fig. 5A–C). Consistent with the previous KEGG results, the major enriched proteins were C4B, PLG, KNG1, C9, F2, CFI, SERPINA1, and C4A. The results of Riddell Nina on HM chicken model showed that complement coagulation cascade and extracellular matrix pathway were overexpressed in HM^13^. It further suggests that the above proteins may be the key factors of pathogenicity.

#### PPI analysis

**Figure 6:**
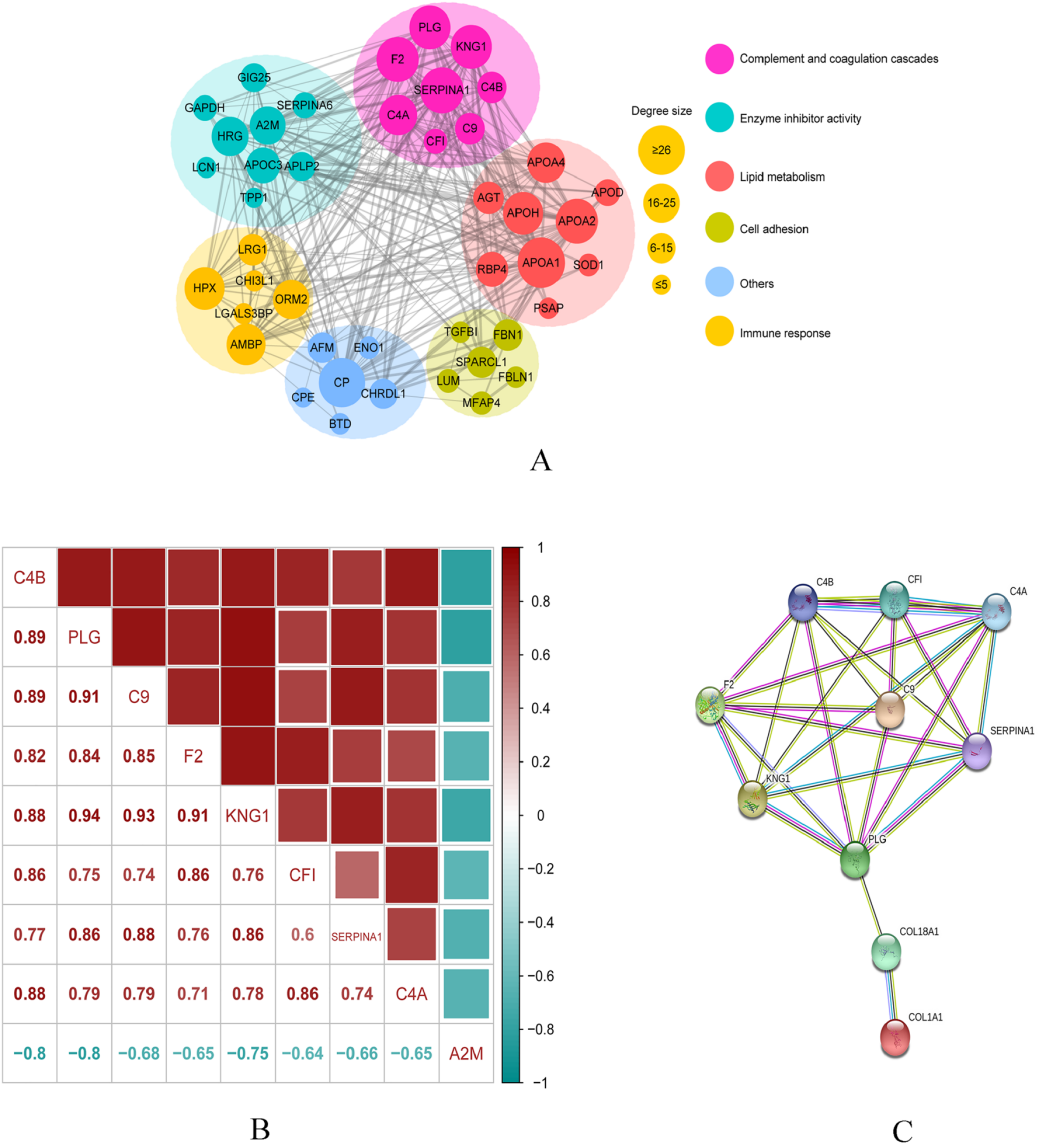
(**A**) Protein–protein interactions of differentially expressed proteins. (**B**) Pearson correlation analysis of the proteins involved in the GSEA complement coagulation cascade. (**C**) Interaction between collagen I, collagen XVIII and the proteins involved in the complement coagulation cascade.

In order to further decipher the potential function of these DEPs, PPIs were analyzed with the STRING database and classified according to DEP functions (information from GO, UniProt, and NCBI). These interacting proteins were divided into six categories, mainly involved in the complement coagulation cascade, enzyme inhibitor activity, lipid metabolism, cell adhesion, and immune response. Figure 6A presents the results.

#### Target protein

Pearson’s correlation analysis of the proteins involved in the GSEA complement coagulation cascade revealed that proteins had a strong correlation in expression (Fig. 6B). Previous studies suggested that collagens I and XVIII played an important role in the pathogenesis of posterior scleral staphyloma caused by HM^14,15^. In the interactions among collagen I, collagen XVIII, and proteins involved in the complement coagulation cascade, SERPINA1, PLG, KNG1, F2, C4A were proteins with high interaction degree (Fig. 6C). Among the five hub DEPs, PLG had the largest expression fold change (7.78), which was equal to the sum of SERPINA1 (2.17), KNG1 (2.59), and F2 (2.99). And PLG is a member of fibrinolytic system, which plays a role in extracellular matrix remodeling and coagulation cascade (Fig. 5C). Therefore, PLG may be the most hub one among them.

#### Validation with ELISA

**Figure 7:**
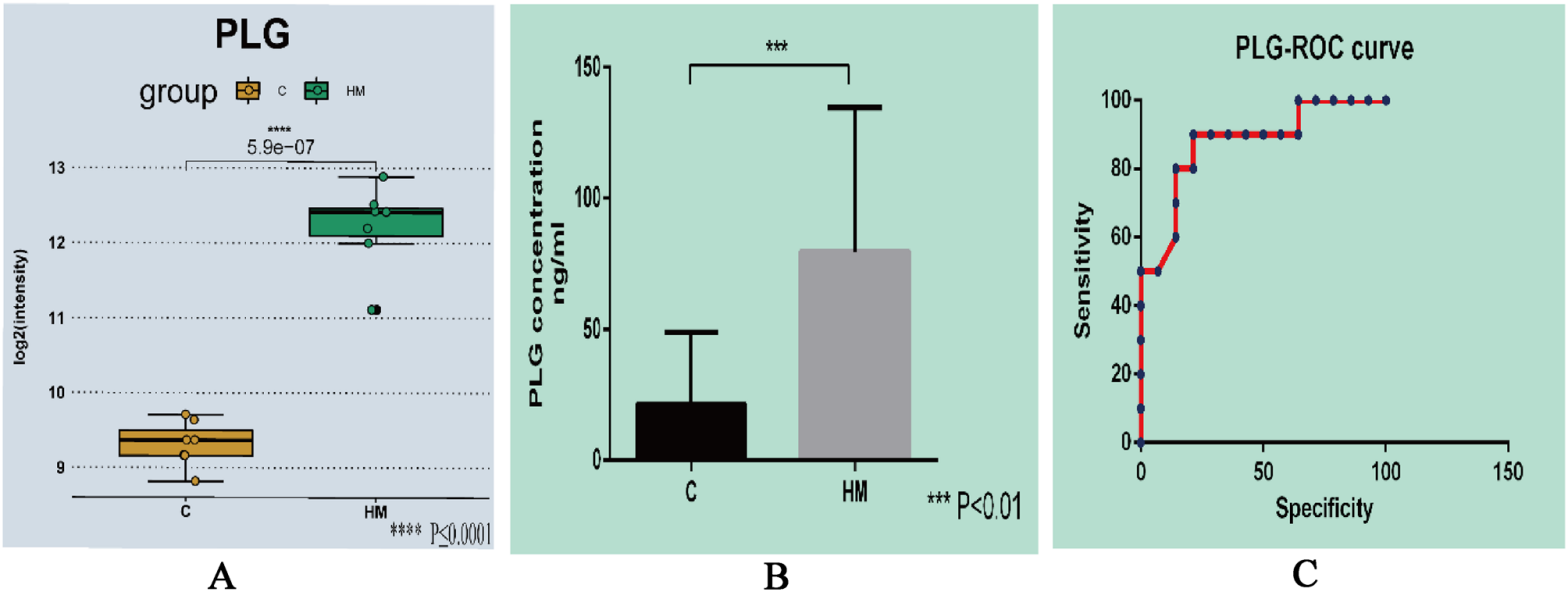
(**A**) A representative MS/MS spectrum indicated important peptide segments for PLG. (**B**) Result of ELISA showed the expression levels of PLG were significantly up-regulated in HM compared to C. (**C**) ROC curve analysis for the specificity and sensitivity of PLG.

We used the ELISA kit to verify the PLG protein expression in the aqueous humor of patients with HM. Figure 7A shows the iTRAQ quantification in the MS/MS spectrum. The expression levels of PLG were significantly upregulated in HM (Fig. 7B), which showed the same trend of change as the proteomic results.

#### ROC curve analysis

The areas under the ROC curves were calculated as measures of the specificity and sensitivity of the confirmed protein (Fig. 7C). The accuracy of the area under the ROC curve was assessed as follows: 0.9–1 = excellent; 0.8–0.9 = good; 0.7–0.8 = fair; and < 0.7 = not useful. The ROC analysis identified that PLG was informative (AUC: 0.8750, 95% confidence interval: 0.7280–1.022).

## Discussion

The development of HM is associated with changes in the collagen fibers of the sclera, as demonstrated in both human and animal models^9,14^. Studies have identified numerous proteins that are involved^9,16,17^. However, to date, most insights regarding HM have come from studies on animal models, which typically focused on the retina, choroid, and sclera^9^. Although changes in protein levels of the aqueous humor in myopic eyes have been extensively studied, few studies have focused on HM. Therefore, the aim of this study was to investigate the changes in aqueous humor protein levels in HM. The results of proteomics might offer new insights to elucidate the mechanism of HM and identify new potential targets for the treatment.

In this study, a total of 211 proteins were identified. The iTRAQ technique revealed 58 DEPs in the aqueous humor of patients with HM, which indicated that the protein expression in the aqueous humor changes in HM. However, the origin and causal relationship with those proteins are unclear. In the study investigating the proteome of the aqueous humor in patients with HM versus controls using iTRAQ-based quantitative proteomics by Xiang M and colleagues, they compared proteins in the aqueous humor of 12 patients undergoing cataract surgery (6 patients with HM, 6 controls) and found 445 proteins including146 DEPs^18^, with no mention of PLG among the significant proteins probably because of sample differences. Duan et al. characterized the aqueous humor of five patients with HM and five age-matched patients with cataract without HM undergoing routine senile cataract surgery and identified that a total of six spots were significantly increased in the two-dimensional gel analysis in HM, with the proteins being albumin, transthyretin, and a vitamin D-binding protein^9^. Transthyretin and the vitamin D-binding protein were included in the 211 proteins we identified but not in the 58 DEPs. This may be caused by differences in protein identification techniques.

The upregulated DEPs were highly concentrated in the coagulation and complement cascade (GSEA and KEGG analysis). A meta-analysis of transcriptome datasets in chick myopia and hyperopia models provided the evidence for involvement of the complement pathway in hyperopia^13^. Some studies showed that the complement system might promote extracellular matrix (ECM) remodeling in the sclera^13,19^.

PLG can be activated by the tissue or urokinase-type plasminogen activator as active plasmin. Plasmin degrades fibrin and converts potential matrix metalloproteinases (pro-MMPs) into active MMPs, which can destroy the ECM^20^. Moreover, plasmin plays an important role in many physiological and pathological processes which may be involved in the pathogenesis of high myopia, such as tissue remodeling, angiogenesis^21–24^. Additionally, some studies have shown that plasmin is closely related to inflammation, autoimmunity, tumor formation, and neurodegeneration^25–27^. Thus, PLG is a multifunctional factor associated with the pathogenesis of HM that warrants further investigations.

Posterior scleral staphyloma is a basic lesion in a series of interrelated degenerative changes in HM and significantly associated with macular holes, retinal detachment, and myopic foveoschisis; therefore, it is recognized as a hallmark of HM^28^. Previous studies pointed out that the overall scleral thinning observed in highly myopic human eyes were associated with a reduction in size of the individual collagen fibrils with a preponderance of unusually small diameter fibrils averaging below 60–70 nm^29^. After 12 days of experimental myopia in the tree shrew (equivalent to 12–14 D myopia), the thickness of the posterior sclera had been reduced by 20% and showed a slow, progressive thinning subsequently. Simultaneously, the dry weights of the sclera and the posterior sclera were reduced by 7% and 17%, respectively^29^. These ultramicroscopic alterations in the sclera of patients with HM suggested that the disorder of the scleral collagen fibrils may be due to the decreased synthesis or increased accentuated breakdown. The overexpression of the PLG protein in HM activates it outside the blood vessels, dissolves the fibers, and makes the sclera thinner, which accelerates the occurrence of posterior scleral staphyloma. According to the website of UniProt (https://www.uniprot.org/), KNG1 plays a role in increasing vascular permeability. Therefore, the upregulated KNG1 can damage the blood–retina barrier and increase vascular permeability by regulating the tight junction with the vascular endothelium, which in turn causes inflammatory mediators to enter the aqueous or vitreous humor. Furthermore, the enriched ECM regulatory factor signaling pathway indicates that the activated PLG may reduce scleral hardness by affecting the composition of the scleral connective tissue. Additionally, the classical signaling pathways of the ECM regulator suggest that excessive activation of PLG may reduce the scleral stiffness by affecting the components of scleral connective tissue. The composition of the scleral ECM is the key to maintain its inherent stiffness, strength, and elasticity.

Apart from its potential role in the mechanism of posterior scleral staphyloma caused by HM, PLG plays a role in the pathogenesis of other complications. First, glaucomatous optic neuropathy (GON) is a common cause of irreversible vision loss worldwide. Several hospital- and population-based investigations showed that the prevalence of GON was higher in highly myopic eyes compared to emmetropic eyes^30–36^. Enlargement of the optic disc, including the stretching and thinning of the sieve plate, may be a factor causing susceptibility to GON^37^. Therefore, we speculated that fibrinolysis induced by PLG activation may be the main factor of optic disc stretching and cribriform plate thinning in HM, which may also be the reason why patients with HM are prone to normal-tension glaucoma^38,39^. Additionally, liquefaction of the vitreous humor begins at a relatively young age in patients with HM^40,41^, which may directly relate to PLG activation. Harocopos and Filas et al. suggested that loss of the vitreous gel with aging increases the risk of nuclear cataracts^42,43^, which are strongly associated with HM^44,45^. Therefore, the activation of PLG may be the cause of patients with HM suffering from nuclear cataracts.

Our study had some limitations. Frist, the sample size was relatively small because of the stringent patient recruitment criteria. Second, we have not conducted further animal and cellular studies on the role of PLG in the pathogenesis of posterior scleral staphyloma. However, the proteomic results were verified using an independent cohort with ELISA (supplementary table).

## Supplementary Information

Supplementary Table
