# Supplementary material for: The plasminogen protein is associated with high myopia as revealed by the iTRAQ-based proteomic analysis of the aqueous humor
Source: Sci Rep. 2021 Apr 22;11:8789. doi: 10.1038/s41598-021-88220-9 (PMC8062568; doi:10.1038/s41598-021-88220-9)
Supplement: Supplementary file 1 — Supplementary Table [file 41598_2021_88220_MOESM1_ESM.docx]

| **Samples** | **gender** | | | **ages** | | **axial** | | **eye** | |
| --- | --- | --- | --- | --- | --- | --- | --- | --- | --- |
|  | **C** | **HM** | **C** | | **HM** | **C** | **HM** | **C** | **HM** |
| **1** | **F** | **F** | **53** | | **68** | **23.68** | **31.66** | **OD** | **OS** |
| **2** | **M** | **F** | **74** | | **61** | **23.27** | **29.21** | **OD** | **OS** |
| **3** | **M** | **F** | **73** | | **63** | **24.46** | **30.30** | **OS** | **OD** |
| **4** | **F** | **M** | **70** | | **60** | **22.78** | **26.19** | **OD** | **OS** |
| **5** | **M** | **F** | **74** | | **70** | **24.16** | **29.45** | **OS** | **OS** |
| **6** | **F** | **F** | **63** | | **60** | **23.06** | **29.19** | **OD** | **OS** |
| **7** | **M** | **M** | **61** | | **63** | **23.10** | **28.71** | **OS** | **OD** |
| **8** | **M** | **F** | **73** | | **62** | **23.56** | **27.81** | **OD** | **OD** |
| **9** | **F** | **M** | **68** | | **61** | **22.25** | **28.92** | **OS** | **OD** |
| **10** | **M** | **F** | **62** | | **54** | **24.67** | **26.41** | **OD** | **OS** |
| **11** |  | **M** |  | | **56** |  | **27.44** |  | **OS** |
| **12** |  | **M** |  | | **64** |  | **31.81** |  | **OD** |
| **13** |  | **F** |  | | **71** |  | **32.45** |  | **OD** |
| **14** |  | **M** |  | | **65** |  | **28.47** |  | **OS** |
| ***P*** |  |  | **0.08** | | | **＜0.01** | |  |  |
| **Mean** |  |  | **67.10±7.09** | | **62.71±4.80** | **23.50±0.76** | **29.14±1.91** |  |  |

Table 1： Patient Characteristics. C: simple nuclear cataract;

HM: nuclear cataract complicated with high myopia；F：female；M：male
